# Editorial Department

**Published:** 1854-01

**Authors:** 


We have received the "Family Dental Journal," edited by D. C. Estes, Dentist, Albany, N. Y.

Dentistry is one of the specialties to which the objections of a previous article do not particularly apply; albeit, we have known a dentist engaged for a week in plugging, pulling, and filing the teeth, in the vain attempt to relieve, thereby, the pains of one who was suffering from inflammation within the antrum of Highmore. We fear, too, that the dental art is guilty of some sins against facial neuralgia.

The "Family Dental Journal" is to diffuse dental information, and urge upon the American people the importance of those "grinders," which they hardly ever use. *Perge et vale!* S. B. H.

